# Gata3 Acts Downstream of β-Catenin Signaling to Prevent Ectopic Metanephric Kidney Induction

**DOI:** 10.1371/journal.pgen.1000316

**Published:** 2008-12-26

**Authors:** David Grote, Sami Kamel Boualia, Abdallah Souabni, Calli Merkel, Xuan Chi, Frank Costantini, Thomas Carroll, Maxime Bouchard

**Affiliations:** 1 Goodman Cancer Centre, McGill University, Montreal, Quebec, Canada; 2 Department of Biochemistry, McGill University, Montreal, Quebec, Canada; 3 Research Institute of Molecular Pathology, Vienna Biocenter, Vienna, Austria; 4 Department of Internal Medicine, University of Texas Southwestern Medical Center at Dallas, Dallas, Texas, United States in America; 5 Department of Molecular Biology, University of Texas Southwestern Medical Center at Dallas, Dallas, Texas, United States in America; 6 Department of Genetics and Development, Columbia University Medical Center, New York, New York, United States in America; Medical Research Council Human Genetics Unit, United Kingdom

## Abstract

Metanephric kidney induction critically depends on mesenchymal–epithelial interactions in the caudal region of the nephric (or Wolffian) duct. Central to this process, GDNF secreted from the metanephric mesenchyme induces ureter budding by activating the Ret receptor expressed in the nephric duct epithelium. A failure to regulate this pathway is believed to be responsible for a large proportion of the developmental anomalies affecting the urogenital system. Here, we show that the nephric duct-specific inactivation of the transcription factor gene *Gata3* leads to massive ectopic ureter budding. This results in a spectrum of urogenital malformations including kidney adysplasia, duplex systems, and hydroureter, as well as vas deferens hyperplasia and uterine agenesis. The variability of developmental defects is reminiscent of the congenital anomalies of the kidney and urinary tract (CAKUT) observed in human. We show that *Gata3* inactivation causes premature nephric duct cell differentiation and loss of *Ret* receptor gene expression. These changes ultimately affect nephric duct epithelium homeostasis, leading to ectopic budding of interspersed cells still expressing the Ret receptor. Importantly, the formation of these ectopic buds requires both GDNF/Ret and Fgf signaling activities. We further identify *Gata3* as a central mediator of β-catenin function in the nephric duct and demonstrate that the β-catenin/Gata3 pathway prevents premature cell differentiation independently of its role in regulating *Ret* expression. Together, these results establish a genetic cascade in which *Gata3* acts downstream of *β-catenin*, but upstream of *Ret*, to prevent ectopic ureter budding and premature cell differentiation in the nephric duct.

## Author Summary

In humans, kidney development originates during embryonic development by the sprouting of an epithelial bud—called the ureteric bud—from a simple epithelial structure—the nephric duct. The ureteric bud quickly grows and branches in a treelike fashion to form the kidney collecting duct system, while the emerging ureteric tips induce nephron differentiation. One of the most important steps during kidney development is the positioning of a single ureteric bud along the nephric duct, since mutations of genes implicated in this process lead to severe urogenital malformations. In this study, we identified the Gata3 protein as a crucial regulator of ureteric bud positioning by using genetically modified mice. Deleting the Gata3 gene in the mouse resulted in the development of multiple kidneys emerging at improper positions. We show that this defect was caused by a hypersensitivity of nephric duct cells in their response to local growth signals. Interestingly, this phenomenon was partly triggered by premature differentiation of a subset of nephric duct cells. Furthermore, we report a genetic pathway in which Wnt/β-catenin signaling activates the *Gata3* gene, which in turn positively regulates the *Ret* gene. In summary, we introduce a mouse model system that can be used to study human birth defects affecting the urogenital system.

## Introduction

In human, urinary tract anomalies rank among the most common birth defects, with an estimated occurrence of 1 in 250 live births [1]. Most of these ontogenic malformations are classified as Congenital Anomalies of the Kidney and Urinary Tract (CAKUT) [2], which is a highly heterogenous condition frequently diagnosed in combination with genital tract anomalies [3]. The most relevant clinical manifestations include absent, dysplastic or obstructed renal systems in infants as well as infertility, pregnancy complications, hypertension and chronic renal failure in adults [4].

The development of the urogenital system (UGS) begins with the formation of the nephric duct (or Wolffian duct) [5],[6]. This epithelial duct is a central UGS component among all vertebrates and serves as the primordium for the ureter, kidney collecting duct system and male genital tract [7]. Upon its induction in the intermediate mesoderm at embryonic day (E) 8.5 in the mouse, the nephric duct rapidly elongates caudally until it reaches the cloaca, a pouch from which the bladder and urethra later develop. At E10.5, the formation of the definitive (metanephric) kidney is initiated by sprouting of the ureteric bud from the nephric duct into the adjacent metanephric mesenchyme. The ureteric bud subsequently undergoes several branching cycles to form the collecting duct system, whereas the ureter tips induce nephron formation in the surrounding mesenchyme [8].

Ureteric bud outgrowth and positioning are among the most crucial steps of UGS development, since anomalies at the budding stage account for the majority of kidney and urinary tract developmental defects [9],[10]. Extensive evidence has identified GDNF/Ret signaling as a central regulator of ureteric bud induction [11]–[16]. In this system, GDNF secretion in the metanephric mesenchyme activates the Ret receptor tyrosine kinase via its ligand binding GFRα1 co-receptor. In turn, Ret activation results in the initiation of intracellular signaling cascades, which mediate bud outgrowth, proliferation and subsequent ureter branching. Understandably, the activity of GDNF/Ret signaling is tightly regulated by various mechanisms to allow for the generation of a single ureter at the appropriate position [9]. In the mesenchyme surrounding the nephric duct, the forkhead transcription factor FoxCI and the Slit2/Robo2 ligand-receptor pair repress the rostral expression of GDNF [17],[18], while Bmp4 antagonizes its activity [19]. At the budding site, Gremlin releases GDNF inhibition by Bmp4 [20], thereby allowing ureteric bud outgrowth. In the nephric duct epithelium, Sprouty1 protein function is crucial to negatively modulate Ret signaling levels [21]. Of notice, gene mutations in any of these regulators of GDNF/Ret signaling result in CAKUT-like phenotypes in mice. In support of the clinical importance of these genes, a growing number of them are found mutated in human developmental diseases affecting the UGS [22]–[26].

Gata3 is a transcription factor of the Gata Zn-finger family, which perform important functions during organogenesis [27]. In humans, *GATA3* haploinsufficiency causes hypoparathyroidism, sensorineural deafness and renal anomalies (HDR) syndrome [28]. The urogenital defects of HDR patients closely resemble CAKUT in combination with genital tract anomalies and include renal aplasia, dysplasia, hypoplasia and vesicoureteral reflux [29],[30]. Gene ablation studies in mice further revealed a critical role for *Gata3* in the development of several tissues [31],[32]. In the urogenital system, *Gata3* is necessary for proliferation control and guidance of the nephric duct [33]. Accordingly, it is the only Gata factor expressed in this tissue prior to E12.5 [34].

Here we report the conditional inactivation of *Gata3* specifically in the nephric duct, at a stage past the developmental defects observed in germline knockout embryos. These mice display multiple UGS malformations affecting the kidney, ureter and genital tracts. The detailed analysis of this phenotype reveals a genetic cascade whereby β-catenin promotes Gata3 expression in the nephric duct, which in turn activates *Ret* expression, maintains an undiffererentiated epithelial cell state and prevents the inappropriate response to signaling pathways promoting ureter budding.

## Results

### Conditional Inactivation of *Gata3* in the Urogenital System

**Figure 1:**
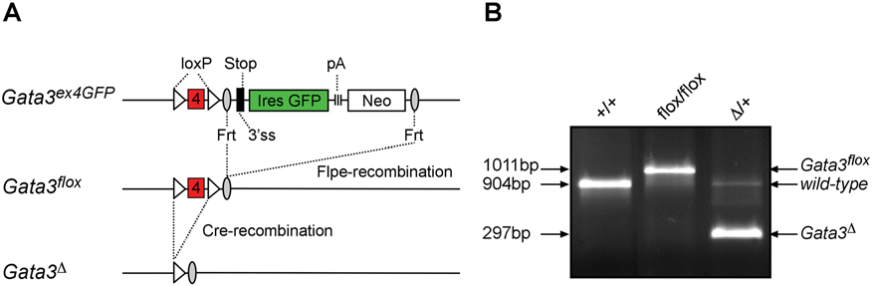
Generation of a conditional *Gata3* knockout allele. (A) The conditional *Gata3* knockout allele was generated by crossing *Gata3^ex4GFP^* mice with *FLPe* germline deleter mice. The FLPe recombinase removed the *Ires-GFP-neo* cassette, thereby generating a *Gata3* allele with *loxP* sites flanking exon 4 (*Gata3^flox^*). Subsequent crosses of *Gata3^flox^* mice with *More-Cre* germline deleter mice resulted in *Gata3*-exon 4 excision and the creation of a null allele (*Gata3^D^*). (B) Representative genotyping by PCR for wild-type (+/+), *Gata3^flox/flox^* (flox/flox) and *Gata3^Δ/+^* (Δ/+) mice prepared from tail DNA.

The strong mesonephric phenotype observed in *Gata3^−/−^* embryos [33] precludes the study of *Gata3* function later during urogenital system development. To investigate the role of *Gata3* in ureteric bud formation, we first generated a *Gata3* conditional loss of function allele (Figure 1A,B). For this, the parental *Gata3* allele (*Gata3^ex4GFP^*) [33] was crossed with a transgenic strain expressing *FLPe* in the germline [35] to excise the *GFP-neo* reporter cassette, thereby generating a conditional *Gata3* allele with loxP sites flanking exon 4 (*Gata3^flox^*). These mice were subsequently bred with the *More-Cre* germline deleter strain [36] to generate the *Gata3^Δ^* allele in which exon 4 is removed. The splicing from exon 3 to exon 5, expected from this modification, would generate a frameshift leading to protein truncation just upstream of the first zinc-finger DNA-binding domain. This gene mutation is therefore predicted to be null, like other previously reported *Gata3* mutant alleles [32],[33],[37]. To delete *Gata3* specifically in the maturing nephric duct and derived collecting duct system, we crossed *Gata3^flox^* mice with the *HoxB7-Cre* mouse strain [38] to generate *HoxB7-Cre*; *Gata3^flox/Δ^* and *HoxB7-Cre*; *Gata3^flox/flox^* embryos. Both genotypes had the same phenotype and are subsequently referred to as *Gata3^ND−/−^* embryos. *Gata3^flox/flox^*, *Gata3^flox/+^* and *HoxB7-Cre*; *Gata3^flox/+^* embryos failed to show any overt phenotype and were used as controls.

### *Gata3^ND−/−^* Embryos Show Multiple Urogenital System Abnormalities

**Figure 2:**
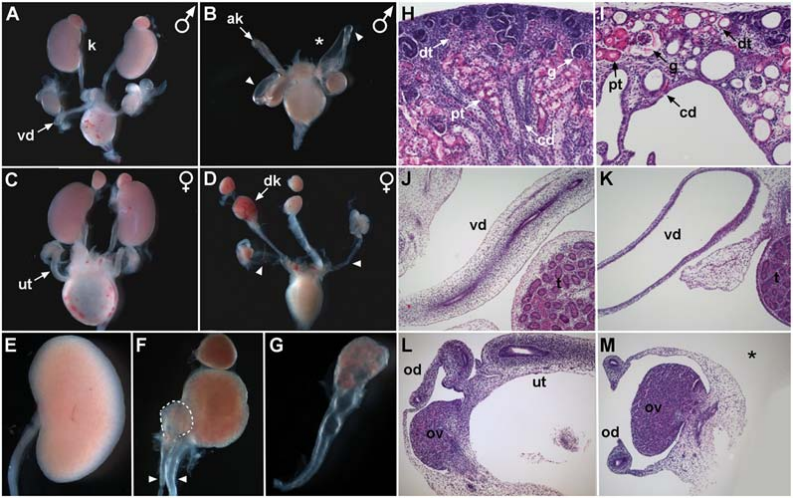
*Gata3^ND−/−^* embryos display multiple urogenital system abnormalities. Urogenital systems were dissected at E18.5 and either processed as whole mount (A–G) or subjected to H&E analysis on tissue sections (H–M). (A) Wild-type male UGS showing normal kidneys (k) and vas deferens (vd). (B) UGS of a *Gata3^ND−/^* ^−^ male embryo, showing unilateral kidney agenesis (*), an aplastic kidney (ak) and bilateral vas deferens dilations (white arrowheads). (C) Wild-type female UGS harboring a normal uterus (ut). (D) Female *Gata3^ND−/−^* UGS, with dysplastic kidneys (dk) and bilateral agenesis of the uterus (white arrowheads point to remaining connective tissue). (E) Wild-type kidney and ureter. (F) Duplex kidneys and ureters (white arrowheads) and (G) hydroureter, hydronephrosis and kidney dysplasia are frequently observed in conditional *Gata3^ND−/^* ^−^ embryos. (H, I) H&E histological analysis of wild-type (H) and *Gata3^ND−/−^* (I) kidneys reveals severe kidney dysplasia and hydronephrosis affecting the collecting duct system and all nephron segments. (J, K) Histological analysis of wild-type (J) and *Gata3^ND−/−^* (K) male genital tracts highlighting the dilated and fluid filled vas deferens in mutant embryos. (L, M) H&E staining of wild-type (L) and *Gata3^ND−/−^* (M) female genital tract, confirming uterus agenesis (*) and showing normal morphology of the ovaries (ov) and oviducts (od) in mutant embryos. cd, collecting duct; dt, distal tubule; g, glomerulus; pt, proximal tubule.

We initially characterized whole dissected urogenital systems (UGS) of *Gata3^ND−/−^* embryos at embryonic day (E)18.5. This gross analysis revealed a broad variety of malformations affecting the kidneys and genital tracts. Kidney defects included agenesis (15%), aplasia (20%) and severe dysplasia (65%) (Figure 2A–G). Moreover, one third of the *Gata3^ND−/−^* embryos displayed duplex kidneys (arrows in 2F). Histological analysis further revealed that the dysplastic kidneys were associated with hydronephrosis and hydroureter (Figure 2H,I). As expected from embryos with such poor kidney endowment, no *Gata3^ND−/−^* pups could be recovered after birth.

In addition to these renal defects, over 80% of male mutant genital tracts displayed a massive enlargement of the vas deferens in comparison to control embryos (Figure 2A,B,J,K). In female embryos, *Gata3* inactivation in the nephric duct resulted in a complete loss of uterus in over 85% of UGSs examined. The oviduct, however, was still present in these embryos (Figure 2C,D,L,M). Hence the conditional inactivation of *Gata3* leads to a broad spectrum of urogenital defects, including a high incidence of hydronephrotic kidneys and hydroureters.

### Multiple Ectopic Buds Are Observed in *Gata3^ND−/−^* Embryos

The combination of kidney hydronephrosis, hydroureter and duplex kidneys seen in *Gata3^ND−/−^* embryos pointed to a primary defect at the level of ureter budding. In order to easily visualize nephric duct cells, we crossed the *Rosa26^STOPLacZ^* allele [38] into control and *Gata3^ND−/−^* genetic backgrounds and stained the UGSs for β-Galactosidase activity.

**Figure 3:**
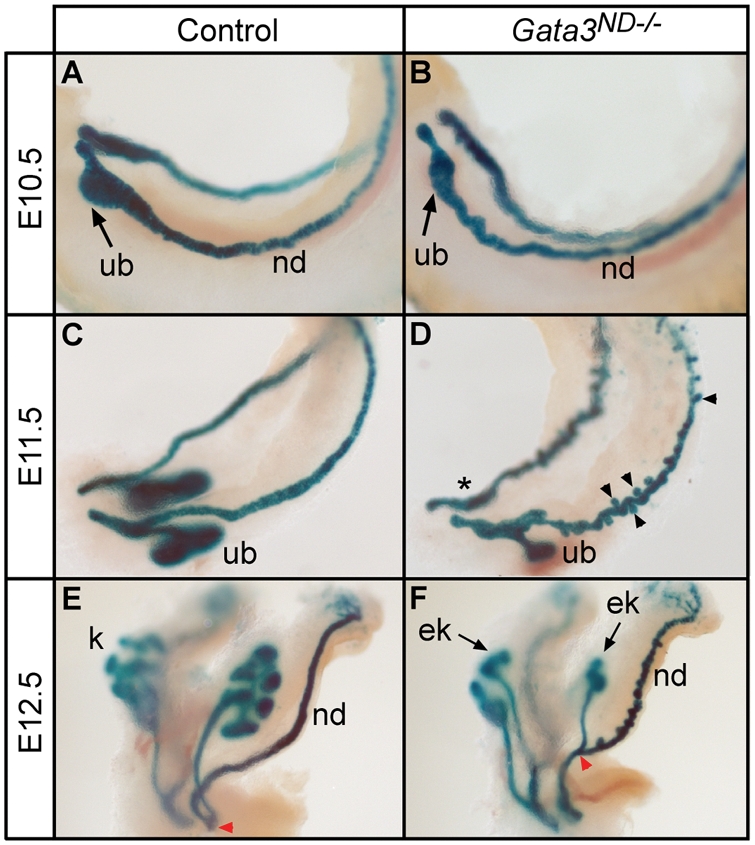
Ectopic ureteric budding in *Gata3^ND−/−^* embryos. (A–F) Dissected urogenital systems of control (*HoxB7-Cre*; *Gata3^flox/+^*; *Rosa26^STOPlacZ/+^*) and *Gata3^ND−/−^* (*HoxB7-Cre*; *Gata3^flox/flox^*; *Rosa26^STOPlacZ/+^*) embryos were stained for β-Galactosidase activity at the indicated embryonic stages. (A, B) Swelling of the caudal portion of the nephric duct (nd) at E10.5 marks the ureteric bud induction site (ub) in control and *Gata3* mutant embryos. (C, D) At E11.5, the ureteric bud of control embryos invades the metanephric mesenchyme to form a T-stage kidney. In *Gata3^ND−/^* ^−^ embryos, ectopic buds form along the entire length of the nephric duct (arrowheads). *Gata3*-deficient embryos frequently fail to induce the primary bud (*). (E, F) At E12.5 the metanephric kidney (k) undergoes its third round of branching morphogenesis in control embryos. In *Gata3^ND−/^* ^−^ embryos, some ectopic buds give rise to ectopic kidneys (ek) showing deficient branching morphogenesis, whereas the most rostral buds start to regress. Note the aberrant branch point of the ectopic kidneys (red arrowhead).

**Figure 4:**
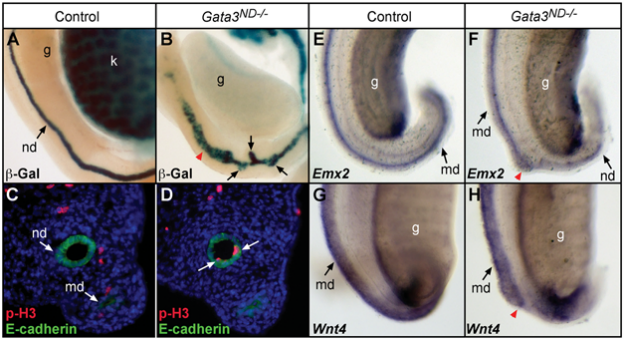
Genital tract anomalies in *Gata3^ND−/−^* embryos. (A, B) β-Galactosidase staining of E14.5 genital ridges marks the beginning of Wolffian duct hyperplasia in male *Gata3^ND−/^* ^−^ embryos (red arrowhead in B). (C, D) Immunostainings for the mitosis marker phosphorylated Histone H3 (p-H3) show an increase in cell proliferation (n = 4, P = 0.09, Student's t-test) in *Gata3^ND−/−^* nephric ducts at E13.5. E-cadherin labeling marks the genital tracts. (E–H) Whole-mount in situ hybridizations for *Emx2* (E, F) or *Wnt4* (G, H) of control and *Gata3^ND−/−^* E13.5 genital ridges reveal an arrest of Müllerian duct elongation (red arrowhead in F, H) in *Gata3^ND−/−^* embryos. Notably, the elongation arrest occurs at the level of persisting ectopic ureteric buds (black arrows in B). nd, nephric duct; md, Müllerian duct; k, kidney; g, gonad.

During normal development, a localized swelling of the caudal portion of the nephric duct indicates the site of ureteric bud outgrowth at E10.5 (Figure 3A). The bud quickly emerges and undergoes the first dichotomous branching event at E11.5, forming the T-stage kidney (Figure 3C). Subsequently, the ureter lengthens and multiple branching cycles mark the development of the metanephric kidney (Figure 3E). In *Gata3^ND−/−^* mutant embryos at E10.5, nephric duct swelling was sometimes observed (Figure 3B), but normal ureteric bud formation failed in most embryos analyzed. In addition, the nephric duct looked more sinuous in appearance (Figure 3B). Strikingly, at E11.5, ectopic epithelial buds formed along the entire length of the nephric duct, with a preferential accumulation in the middle segments of the duct (Figure 3D). By E12.5, the majority of the ectopic buds started to regress, while some buds expanded to form ectopic kidneys at a position far more rostral than the normal kidney induction site (Figure 3F). Following ectopic bud regression, the male nephric duct started to enlarge (Figure 4A,B) This was accompanied by a slight increase of about 50% in cell proliferation index, as determined by phospho-histone H3 immunolabelling at E13.5 (Figure 4 C,D). In females, in situ hybridization with cRNA probes against *Emx2* and *Wnt4*, staining the Müllerian duct epithelium and mesenchyme, respectively, revealed a block in female genital tract elongation in the region where most ectopic ureteric buds occurred (Figure 4E–H). Since the expression of the key Müllerian duct regulators *Wnt4*, *Emx2*, *Wnt9b* and *Lim1* was normal in these embryos (Figure 4E–H and data not shown), it is possible that this elongation defect is simply caused by a physical obstruction by ectopic ureteric buds. Together, these results indicate that the urogenital defects observed in *Gata3^ND−/−^* embryos are largely caused by the emergence of ureteric buds at aberrant positions along the nephric duct.

### The Formation of the Ectopic Buds Depends on GDNF/Ret as well as Fgf Signaling

**Figure 5:**
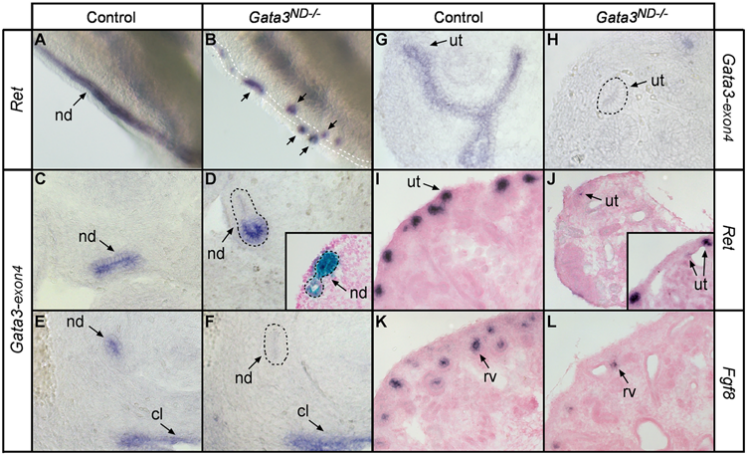
Gata3 is necessary for maintenance of *Ret* expression. (A–F) Gata3 maintains *Ret* expression in the nephric duct at E11.5. (A) Whole mount in situ hybridization of control UGS with a *Ret* cRNA probe shows the smooth gradient of *Ret* expression along the entire length of the nephric duct. (B) In *Gata3^ND−/−^* embryos, *Ret* expression is lost in most nephric duct cells except for some cells located in the ectopic ureteric bud tips (arrows). (C,D) In situ hybridizations with a *Gata3* cRNA probe specific for exon 4 confirm that the *Ret* expressing cells in the ectopic buds still express *Gata3*. In these embryos, *Gata3*+ cells segregate from *Gata3*− cells. A similar tendency is observed in *Gata3^ND−/−^* embryos stained for β-Galactosidase activity (D insert). (E, F) The caudal nephric duct efficiently deleted *Gata3* already at this stage. (G–L) Deficient branching morphogenesis and nephron differentiation in *Gata3^ND−/−^* hypodysplastic kidneys. (G,H) In situ hybridization with the *Gata3-exon 4* cRNA probe confirms *Gata3* inactivation in *Gata3^ND−/−^* metanephric kidneys. (I, J) *Ret* expression becomes strongly downregulated in highly dysplastic *Gata3*-mutant kidneys, in contrast to control kidneys. (J insert) Milder kidney dysplasia showing a pattern of *Ret* expression intermediate between wild-type and highly dysplastic kidneys. (K,L) *Fgf8* in situ hybridizations reveal impaired nephron induction in *Gata3^ND−/−^* kidneys. Tissue sections in D (insert) and I–L are counterstained with nuclear fast red. nd, nephric duct; cl, cloaca; ut, ureter tip; rv, renal vesicle.

To determine the cause of ectopic ureter budding in *Gata3^ND−/−^* embryos, we first looked at *Ret* expression by in situ hybridization. Consistent with the previous analysis of *Gata3* germline knockout embryos [33], most *Gata3^ND−/−^* nephric duct cells lost *Ret* expression. Curiously, however, the ectopic buds of *Gata3^ND−/−^* embryos remained positive for *Ret* (Figure 5A,B). In situ hybridization with a cRNA probe against *Gata3* exon 4 (which is excised in the Gata3 conditional allele) confirmed that the bud cells still expressed *Gata3* (Figure 5C,D), whereas nephric duct cells had already lost *Gata3* expression (Figure 5C–F). The remaining *Ret* expression therefore resulted from the incomplete action of Cre in the nephric duct at this stage. Interestingly, these ectopic buds consisted of Gata3+/Ret+ and Gata3−/Ret− cells segregated from each other (Figure 5A–D). The continuous inactivation of *Gata3* as well as the downregulation of *Ret* expression was confirmed in dysplastic *Gata3^ND−/−^* metanephric kidneys derived from such ectopic buds (Figure 5G–J). The severity of kidney dysplasia in these embryos correlated well with the amount of remaining *Ret* expression (Figure 5J insert, data not shown). As expected, these dysplastic kidneys additionally showed severely impaired nephron differentiation, as evidenced by the strong reduction in *Fgf8*-positive nephron precursors in comparison to control embryos (Figure 5K,L).

**Figure 6:**
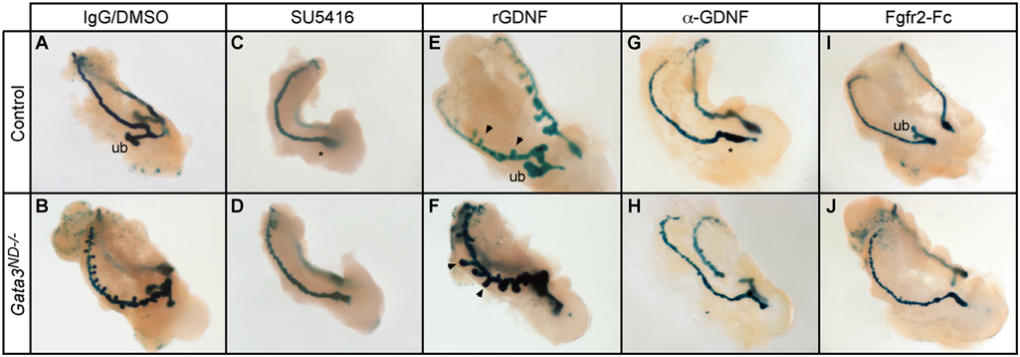
Ectopic ureter budding in *Gata3^ND−/^* ^−^ embryos depends on GDNF/Ret and Fgf signaling. (A, B) E10.5 UGS organ cultures recapitulate the phenotype of both control and *Gata3^ND−/^* ^−^ embryos observed *in vivo*. (C, D) Treatment of cultures with the Ret kinase inhibitor SU5416 efficiently inhibits both primary (*) and ectopic budding in control and *Gata3^ND−/^* ^−^ UGSs. (E, F) Supplementing the organ cultures with recombinant GDNF induces ectopic ureteric budding in control UGS cultures (arrows in E) and enhances ectopic bud growth in *Gata3^ND−/^* ^−^ UGS cultures (arrows in F). (G, H) Organ culture of control or *Gata3*-deficient UGSs treated with a GDNF blocking antibody suppresses primary and ectopic bud formation, respectively. (I, J) Soluble FgfR2-Fc fusion protein has no major effect on primary budding in control UGSs, but successfully inhibits ectopic budding in *Gata3^ND−/^* ^−^ UGSs. ub, ureteric bud; *, primary bud agenesis.

Since *Ret* expression was specifically localized in the ectopic ureteric buds of *Gata3^ND−/−^* embryos (Figure 5A,B), we hypothesized that Ret signaling might be causally involved in the budding process. To investigate whether the ectopic buds required GDNF/Ret signaling, we performed organ culture experiments with dissected *Gata3^ND−/−^* and control UGSs starting at E10.5. After 42 hours in culture, their development progressed, forming either T-stage metanephric kidneys in control UGSs or multiple ectopic buds in *Gata3^ND−/−^* UGSs (Figure 6A,B). Interestingly, addition of the Ret tyrosine kinase inhibitor SU5416 [39] efficiently suppressed ectopic budding in *Gata3^ND−/−^* UGSs in culture (Figure 6D) as well as primary ureteric bud formation in control UGSs (Figure 6C,D). To further assess the role of GDNF/Ret signaling in ectopic bud formation, we treated cultured UGSs with recombinant GDNF [40]. This treatment, sufficient to induce ectopic budding in control UGS cultures (Figure 6E), significantly increased the size of ectopic buds in *Gata3*-deficient cultures (Figure 6F, compare with 6B). In addition, an antibody blocking GDNF activity successfully inhibited both primary and ectopic bud formation in control and *Gata3^ND−/−^* UGS cultures, respectively (Figure G,H), thereby demonstrating the direct implication of GDNF/Ret signaling in ectopic ureteric bud formation. Interestingly, a 50% lower concentration of GDNF blocking antibody still inhibited primary bud induction but failed to inhibit the formation of ectopic buds (data not shown), suggesting the presence of additional factors involved in the budding process. One candidate is the Fgf signaling pathway, which has the potential to induce ectopic ureter budding [41],[42]. Supplementing the culture medium with a soluble FgfR2-Fc chimeric protein [43] did not perturb primary ureteric bud outgrowth in control cultures (Figure 6I). In striking contrast, however, it could efficiently suppress ectopic ureter budding in *Gata3^ND−/−^* cultures (Figure 6J). In conclusion, these results demonstrate that the generation of the ectopic buds in *Gata3^ND−/−^* embryos is mediated through the combined action of GDNF and Fgf signaling activities.

### Molecular Characterization of Ectopic Ureteric Bud Formation in *Gata3^ND−/−^* Embryos

**Figure 7:**
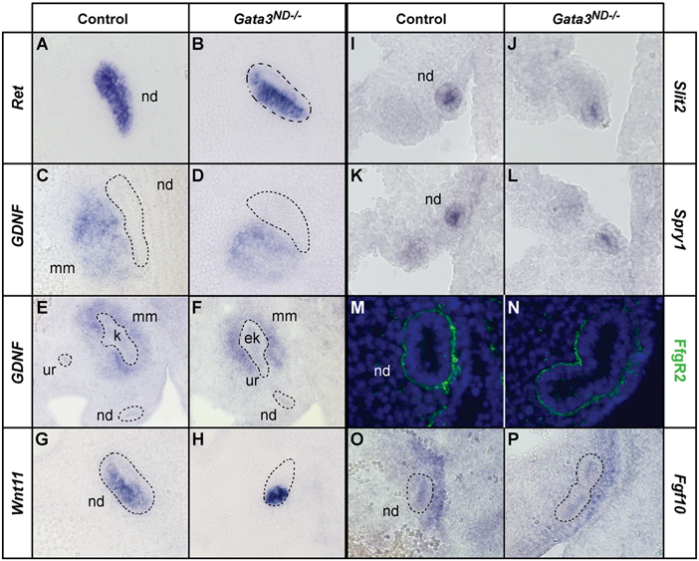
Molecular characterization of ectopic ureteric bud formation in *Gata3^ND−/−^* embryos. (A, B) At E10.5 in *Gata3^ND−/−^* embryos, the Ret+ nephric duct cells (nd) segregate from Ret− cells prior to budding. (C,D) Adjacent sections show that Ret+ cells are localized next to *GDNF* expressing cells in the metanephric mesenchyme (mm). (E, F) Ectopic kidneys (ek) maintain mesenchymal *GDNF* expression in the metanephric mesenchyme at E11.5. (G, H) Induced ectopic ureteric buds in *Gata3*-deficient embryos strongly upregulate the expression of *Wnt11* at E10.5. (I–L) *Slit2* and *Spry1* are slightly downregulated in *Gata3* mutant (nd) cells at E10.5. (M,N) FgfR2-Fc staining shows a strong but unaffected expression of Fgf ligands in both control and *Gata3^ND−/−^* embryos at E11.5. (O,P) *Fgf10* is expressed in the mesenchymal cells adjacent to the nephric duct in both wild-type and *Gata3^ND−/−^* embryos. nd k: kidney, ur ureter.

To study ectopic bud formation at the molecular level, we looked at the expression of key components of the GDNF/Ret signaling cascade in E10.5 and E11.5 embryos. Already at E10.5, prior to budding, *Ret* expression was lost in a subset of nephric duct cells in *Gata3^ND−/−^* embryos (Figure 7A,B). Staining of adjacent sections with a *GDNF* in situ probe revealed that the remaining *Ret*-expressing cells were concentrated near the mesenchymal source of GDNF (Figure 7C,D). At E11.5, only the caudal ectopic buds that could maintain *GDNF* and *Pax2* expression in the surrounding mesenchyme were able to develop into ectopic kidneys (Figure 7E,F and data not shown). In some *Gata3^ND−/−^* embryos, the forming buds induced more *Wnt11* expression than the presumptive ureteric bud induction site in control embryos, pointing to an elevated GDNF/Ret signaling response in those cells (Figure 7G,H). By E12.5, the expression of *Wnt11* and *Ret* was lost in the regressing ectopic buds of *Gata3^ND−/−^* embryos (data not shown). Surprisingly, assessing the expression of the GDNF/Ret modulators *Spry1*, *Spry2*, *Spry4*, *Slit2*, *Robo2*, *FoxcI*, *FoxcII*, *Bmp4* and *Grem1*, only revealed a modest downregulation of *Spry1* and *Slit2* expression in *Gata3-*mutant nephric duct cells at E10.5, while the other markers remained unaffected (Figure 7I–L and data not shown). In order to further support the involvement if Fgfs in ectopic bud formation, we additionally probed control and *Gata3^ND−/−^* embryos with the soluble Fgfr2-Fc fusion protein, which recognizes several Fgfs [44]. This experiment revealed strong and similar Fgf expression levels surrounding the nephric duct in both *Gata3*-mutant and control embryos at E11.5 (Figure 7M,N). This protein localization was consistent with the expression of one of the known FgfR2 ligands, *Fgf10* (Figure 7O,P). Hence, the molecular marker analysis reveals no obvious mesenchymal defects, pointing to a primary defect in the response of the nephric duct to GDNF and Fgf signals.

### β-Catenin Signaling Regulates *Gata3* Expression in the Nephric Duct

**Figure 8:**
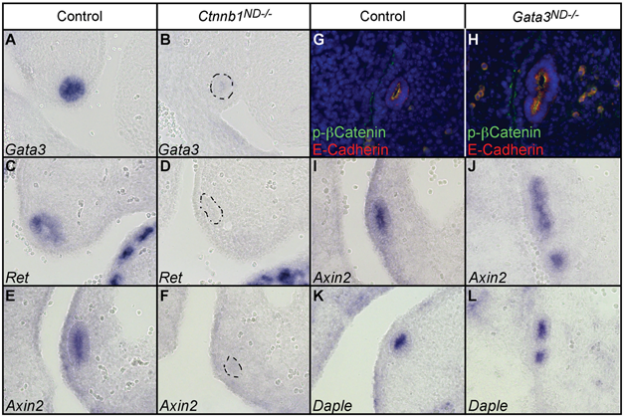
β-catenin regulates *Gata3* expression in the nephric duct. (A, B) In situ hybridizations with a *Gata3* cRNA probe reveal a strong downregulation of *Gata3* expression in the nephric duct of E11.5 *Ctnnb1^ND−/−^* embryos. (C, D) Likewise, in situ hybridizations with a *Ret* probe show a strong decrease of *Ret* expression in *Ctnnb1*-deficient nephric ducts. (E, F) Adjacent sections stained for the β-catenin transcriptional target *Axin2*, confirm loss of β-catenin signaling in *Ctnnb1^ND−/−^* embryos. (G,H) Phosphorylated β-catenin levels are not significantly perturbed in *Gata3^ND−/−^* embryos. E-cadherin was used as a nephric duct marker. (I–L) In situ hybridizations for *Axin2* (I, J) and *Daple* (K, L) show a normal β-catenin signaling response in *Gata3^ND−/−^* nephric ducts.

**Figure 9:**
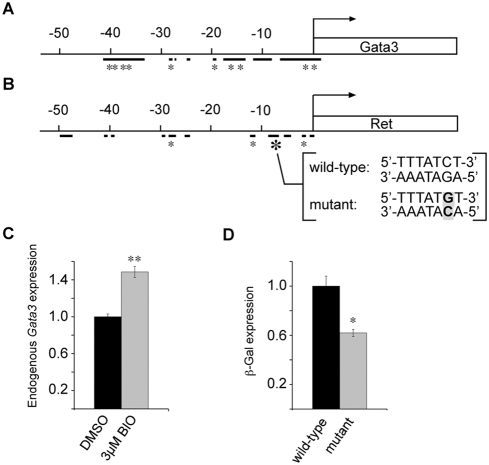
In vitro analysis of the *β-catenin*-*Gata3*-*Ret* molecular cascade. (A, B) Bioinformatic analysis of *Gata3* and *Ret* genomic loci 50 kb upstream of the translational start site. Conserved regions between mouse and human sequences are depicted as thick black lines. Conserved TCF/Lef binding sites (consensus sequence (C/G)TTG(A/T)(A/T)) [69] (A) and Gata3 binding sites (consensus sequence (A/T)GATA(A/T)) [70] (B), are represented as asterisks. (B) A putative Gata3 binding site located in a previously reported *RET* regulatory element [50] and the mutated version used in (D) are indicated in brackets. (C) Quantitative RT-PCR analysis of *Gata3* expression in mIMCD3 reveals a marked increase in endogenous *Gata3* expression upon treatment with 3 µM BIO versus DMSO control. Results are expressed as ratios relative to control (n = 3, ** P<0.01, Student's t-test). (D) β-Galactosidase reporter activity is decreased in Gata3-expressing mIMCD3 cells transfected with an expression construct containing the mutated Gata3 binding sequence (as indicated in B), when compared to the wild-type control. Results are expressed as ratios relative to control. (n = 3, * P<0.05, Student's t-test).

The developmental defects of *Gata3^ND−/−^* embryos described above show striking similarities with the phenotypes resulting from the conditional inactivation of *β-catenin* (*ctnnb1*) in the nephric duct [45]. The fact that both animal models have the same spectrum of genital tract and kidney defects, prompted us to verify whether *Gata3* and *β-catenin* act in the same genetic pathway. For this, we first performed in situ hybridization against *Gata3* in E11.5 *HoxB7-Cre*; *Ctnnb1^flox/−^* embryos (*Ctnnb1^ND−/−^*) and found a strong downregulation of *Gata3* expression in the nephric duct in comparison to control embryos (Figure 8A,B). Accordingly, in situ hybridizations with a *Ret* cRNA probe revealed a loss of *Ret* expression in *Ctnnb1^ND−/−^* embryos (Figure 8C,D), which mimics the loss of *Ret* expression in *Gata3^ND−/−^* embryos (Figure 5). Staining of adjacent tissue sections with in situ probes for the canonical Wnt target genes *Axin2*, *Sp5* and *Daple* [46],[47] confirmed the loss of β-catenin transcriptional response in *Ctnnb1^ND−/−^* embryos (Figure 8E,F and data not shown). In order to clarify the genetic hierarchy between *Gata3* and *β-catenin*, we next stained *Gata3^ND−/−^* embryos with antibodies against β-catenin and phospho-β-catenin. These experiments failed to show any modification of β-catenin expression levels or activity following *Gata3* inactivation (Figure 8G,H and data not shown). In support of this, the expression of the canonical Wnt-signaling target genes *Axin2*, *Daple* and *Sp5* was unchanged in *Gata3^ND−/−^* embryos (Figure 8I–L and data not shown). From these data, we conclude that *Gata3* acts genetically downstream of *β-catenin* but upstream of *Ret* in the nephric duct. To further characterize the molecular basis of the *β-catenin*-*Gata3-Ret* pathway, we first performed a bioinformatics analysis of the 50 kb genomic region upstream of the mouse and human *Gata3* genes. Highly conserved sequences shared by the two species were further analyzed for putative binding sites for TCF/Lef. In total, 10 putative TCF/Lef binding sites, located in 5 of the 8 conserved regions, were identified (Figure 9A). In this analysis, we additionally included a *Gata3* urogenital enhancer located at −110 kb [48], but failed to detect any conserved TCF/Lef binding sites in this element. A similar bioinformatics analysis of the *Ret* regulatory region revealed putative Gata3 binding sites in 4 of the 11 conserved regions 50 kb upstream of the *Ret*-ATG. (Figure 9B). The potential of β-catenin to regulate endogenous *Gata3* expression was further evaluated in mouse IMCD3 collecting duct-derived cells. Using the GSK3β inhibitor BIO to stabilize the β-catenin protein [49], we observed a significant increase of *Gata3* expression levels in those cells (Figure 9C). In order to assess the activity of Gata3 on the *Ret* regulatory region, we took advantage of the fact that one of the Gata3 binding sites mapped to a region previously reported to drive reporter gene expression in the zebrafish pronephros (Figure 9B, large asterisk) [50]. To test whether Gata3 acts on this site, we isolated the 1.2 kb conserved fragment and introduced a specific point mutation in the Gata3 binding site (Figure 9B). The wild-type and mutated fragments cloned upstream of a β-Gal reporter construct were transfected in IMCD3 cells stably expressing Gata3. The inactivation of the Gata3 binding site led to a significant downregulation of β-Gal expression relative to the wild-type control (Figure 9D), thereby suggesting that Gata3 may regulate *Ret* expression directly from this binding site. Together these data are consistent with a *β-catenin*-*Gata3*-*Ret* genetic cascade in the nephric duct.

### Premature and *Ret*-Independent Differentiation of *Gata3* Mutant Cells

**Figure 10:**
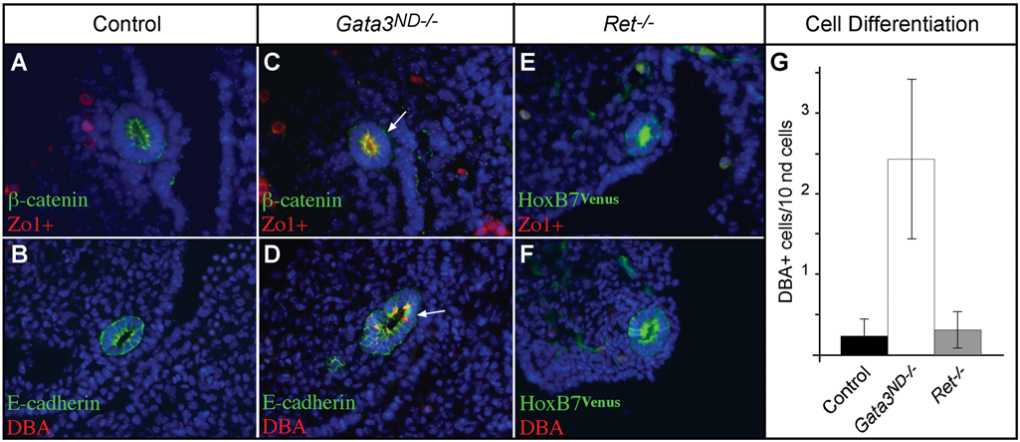
*Gata3* regulates cell differentiation in a Ret independent manner. (A,C) Co-immunostainings for β-catenin and Zo1+ reveal an increase of Zo1+ expression in *Gata3*-deficient nephric ducts at E11.5. (B, D) Similarly, E-cadherin/DBA co-labeling shows stronger DBA staining in *Gata3^ND−/−^* embryos. (E,F and A,B) No change in Zo1+ or DBA expression was observed between *Ret^−/−^* and control embryos at E11.5. The *HoxB7*-driven Venus signal outlines the nephric duct. (G) Quantification of the DBA positive cells reveal a tenfold increase of DBA+ cells in *Gata3^ND−/−^* embryos when compared to control and *Ret^−/−^* embryos (n = 4, P<0.001, Student's t-test).

Another aspect of *β-catenin*-loss in the nephric duct is a premature differentiation of the affected cells [45]. To test whether this phenotype was also present in *Gata3^ND−/−^* embryos, we stained mutant and control embryos with the differentiation markers DBA and Zo1+. In the developing metanephric kidney, DBA and Zo1+ are strongly expressed in the distal collecting duct and downregulated in Ret+ ureter tip cells [45],[51]. At E11.5, DBA and Zo1+ expression were almost undetectable in the nephric duct of control embryos (Figure 10A,B). However, *Gata3*-deficient cells upregulated both differentiation markers already at this stage, suggesting a premature acquisition of a collecting duct identity in these cells (Figure 10C,D). To clarify whether this premature differentiation was a result of *Gata3* inactivation only or secondary to the loss of *Ret* expression in *Gata3*-mutant cells, we next tested the expression of the two differentiation markers in *Ret* mutant embryos. Interestingly, no difference in DBA and Zo1+ expression levels could be observed between wild-type and *Ret* deficient embryos (Figure 10A,B and E,F). To quantifiy the differentiation defect in *Gata3^ND−/−^* embryos, we counted the total amount of DBA-positive nephric duct cells in the three different genotypes. Strikingly, we observed a ten-fold increase in differentiated nephric duct cells in *Gata3-*deficient embryos when compared to control or *Ret* mutant embryos (Figure 10G). From these data, we conclude that Gata3 acts independently of Ret to maintain a precursor state in the nephric duct epithelium, downstream of β-catenin.

## Discussion

We previously reported the critical role played by Gata3 in proliferation control and nephric duct guidance in the pro/mesonephros [33]. To circumvent this early renal phenotype and the embryonic lethality of *Gata3^−/−^* embryos at midgestation [31]–[33], we generated a conditional knockout allele of *Gata3*. In order to specifically address the later role of Gata3 in urogenital system morphogenesis, we inactivated *Gata3* in the nephric duct using the *HoxB7-Cre* transgenic line (*Gata3^ND−/−^*). This resulted in severe malformations of the urogenital system including kidney agenesis, aplasia, dysplasia, duplex systems, uterine agenesis and vas deferens hyperplasia. This spectrum of malformations overlaps with the urogenital phenotypes observed in HDR syndrome patients (heterozygous for *GATA3*) and is generally reminiscent of human congenital anomalies of the kidney and urinary tract (CAKUT). Interestingly, most of these phenotypes can be either directly or indirectly attributed to ectopic ureteric budding observed in *Gata3^ND−/−^* embryos. Furthermore, our results identify *Gata3* as a critical mediator of β-catenin signaling, which regulates both cell differentiation and *Ret* expression in the nephric duct.

### Regulation of *Gata3* by β-Catenin

**Figure 11:**
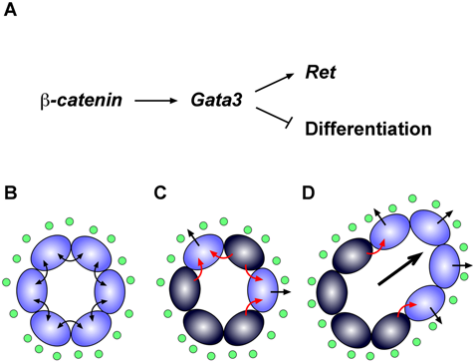
Summary of Gata3 function in progenitor cell state maintenance in the nephric duct. (A) Genetic regulation experiments with *Gata3*, *β-catenin* and *Ret* mutant embryos identify a cascade whereby *Gata3* acts downstream of the *β-catenin* developmental program to independently maintain *Ret* expression and to prevent premature differentiation of nephric duct cells. (B–D) Panel B- In wild-type nephric duct cells, high levels of Gata3 expression maintain nephric duct homeostasis. Panel C- A critical downregulation in Gata3 expression levels (dark blue cells) in *Gata3^ND−/−^* mice (and possibly also in HDR-patients) causes an imbalance in the response to local growth factors such as GDNF and Fgfs (green circles). This may come as a result of premature cell differentiation and differential cell adhesion properties of *Gata3*-deficient cells. Panel D- Consequently, cells with sufficient levels of Gata3 (and Ret) segregate from *Gata3*-mutant cells and expand, thereby forming ectopic buds and kidneys.

The UGS malformations observed in *Gata3^ND−/−^* embryos were strikingly similar to the ones reported recently for *Ctnnb1^ND−/−^* embryos [45]. Gene expression analyses in both genotypes revealed a genetic cascade whereby *Gata3* acts downstream of *β-catenin* in the nephric duct to maintain *Ret* expression and prevent premature epithelial differentiation. These genetic interactions were further supported by promoter analyses and cell culture assays. Hence, our results identify Gata3 as a crucial mediator of β-catenin activity in the nephric duct. Using *Ret^−/−^* embryos, we could further determine that the premature differentiation phenotype observed in *Ctnnb1* and *Gata3* mutant embryos is not mediated by Ret, thereby establishing at least two distinct cellular functions regulated by the β-catenin/Gata3 pathway (Figure 11A). The possibility remains that β-catenin also has a Gata3-independent effect on *Ret* expression. However, our data indicate that, if present, this effect is not sufficient for *Ret* expression in the absence of *Gata3*. The identification of β-catenin acting upstream of *Gata3* also raises the question of the transcriptional control of *Gata3* expression in the nephric duct. The only other regulators of *Gata3* identified in this tissue are the transcription factors Pax2 and Pax8 [33]. It is thus possible that Pax2/8-mediated activation of *Gata3* acts through the β-catenin pathway. Alternatively, Pax2/8 and β-catenin may act independently to regulate *Gata3* expression in the nephric duct either together or as activation and maintenance factors, respectively. We favor the latter model based on the observation that the canonical Wnt signaling readouts *Axin2* and *Daple* are not yet expressed in 18-somite stage embryos (E9.0; D.G., M.B. unpublished results), whereas *Gata3* is already under the control of *Pax* genes at this stage [33]. The recent identification of a *Gata3* kidney enhancer active in the nephric duct may help clarify some aspects of *Gata3* regulation in this tissue [48].

### Mechanisms of Ectopic Ureteric Bud Formation

Defects in ureteric budding account for most UGS defects in humans and mice. The underlying cause is typically a deregulation of GDNF/Ret signaling [9]. Accordingly, our data show a direct implication of GDNF/Ret signaling in the formation of *Gata3^ND−/−^* ectopic buds. Recombinant GDNF treatment indeed sustained ectopic bud growth in organ culture, while blocking the signaling pathway with a GDNF blocking antibody or a chemical inhibitor against Ret was sufficient to prevent ectopic ureter budding. We also demonstrated that the emerging buds consist of Ret+ cells, which respond to mesenchymal GDNF by upregulating *Wnt11* expression. Importantly, however, we show that *Ret* expression was lost in *Gata3*-mutant nephric duct cells and that these Gata3−/Ret− cells failed to contribute to ectopic ureteric bud formation. This suggests that ectopic ureteric buds form as a consequence of *Gata3* loss in neighboring nephric duct cells. It further implies that Ret-mediated signaling played a role in the segregation of Gata3+/Ret+ from Gata3−/Ret− cells. Interestingly, a biased contribution of Ret+ and Ret− cells was also observed in the ureteric bud and metanephric kidney of embryos chimeric for the *Ret* gene [40]. However, the precise mechanism leading to Gata3+/Ret+ and Gata3−/Ret− cell segregation remains unclear.

Importantly, a number of observations argue against a simple GDNF/Ret-based budding mechanism. Among them are 1) the different sensitivity of ectopic buds towards GDNF-blocking antibody concentration and 2) the fact that ectopic buds emerge in all directions whereas GDNF is expressed in the inner intermediate mesoderm only. Together, these observations point to the existence of a parallel pathway to GDNF/Ret promoting ectopic ureter budding in *Gata3^ND−/−^* embryos. Using a soluble recombinant Fgf receptor 2, we identified Fgf signaling as a crucial component of this alternative pathway. In support of this, Fgfs are able to induce ectopic budding in organ culture [41],[42]. Of notice, the RTK inhibitor (SU5416) we used to inhibit Ret signaling in culture was also reported to have an effect on FgfRs [39], which may have enhanced its activity against ectopic ureteric bud formation.

The genetic regulation of *Ret* by Gata3 corroborates our previous observations in germline *Gata3* mutant embryos [33], but raises the intriguing question of the relationship between Gata3+/Ret+ budding cells and their surrounding Gata3−/Ret− cells in ectopic ureteric bud formation. Remarkably, the expression of the GDNF/Ret signaling regulators, *Foxc1*, *Foxc2*, *Robo2*, *Bmp4*, *Grem1 and GDNF* was not significantly affected in *Gata3^ND−/−^* embryos. The modest dowregulation of *Spry1* and *Slit2* expression was additionally restricted to non-budding *Gata3*-mutant cells and might be secondary to the loss of *Ret* expression. Hence, none of the major known mechanisms of GDNF/Ret modulation are likely to cause ectopic ureter budding in *Gata3^ND−/−^* embryos. Instead, this suggests that the juxtaposition of nephric duct cells with different Gata3/Ret status is central to the ectopic budding phenotype. Hence, the simplest model to explain ectopic ureteric bud formation would be that the combined expression of GDNF and Fgf along the defective *Gata3^ND−/−^* nephric duct at E10.5 is sufficient to initiate ectopic bud formation by wild-type nephric duct cells. At E11.5, the source of GDNF becomes restricted to the metanephric mesenchyme adjacent to the caudal region of the nephric duct. Thus, only the ectopic buds which are close enough to this mesenchymal source of GDNF and still harbor a sufficient number of Ret+/Gata3+ cells will be able to sustain the GDNF/Ret/Wnt11 positive feed back loop [52] and continue to grow to form ectopic kidneys. At this stage, most of the rostral buds are devoid of local GDNF expression and will therefore regress.

Importantly, the trigger for the aberrant responses to GDNF and Fgf inductive signals necessarily results from the loss of *Gata3* in the surrounding nephric duct cells (Figure 11B). Ectopic budding may thus be a consequence of the premature differentiation and changes in adhesive properties observed in *Gata3*-mutant cells, as reported for *Ctnnb1^ND−/−^* embryos [45]. Alternatively, the loss of Gata3 may result in the activation of yet unidentified signals affecting the behavior of neighboring cells (Figure 11B). By avoiding these aberrant cellular responses in wild-type embryos, Gata3 maintains epithelial homeostasis and thus prevents the formation of ectopic buds by GDNF and Fgf in nephric duct regions that normally express their respective receptor.

### Gata3 and HDR Syndrome

In humans, heterozygous inactivation of the *GATA3* gene is responsible for HDR syndrome [28],[30]. As many as 35 of the 38 mutations identified to date result in a loss of GATA3 DNA binding activity, mostly as a consequence of complete gene deletion or truncation of GATA3 DNA-binding domains [53]. As in *Gata3^ND−/−^* embryos, this *GATA3* haploinsufficiency in HDR patients leads to a variety of UGS defects such as kidney agenesis, dysplasia, hypoplasia, sometimes associated with female genital tract malformations [29],[30]. This suggests that a critical threshold level of GATA3 is necessary to perform its normal function in the UGS and that cells or tissues expressing GATA3 below this level behave aberrantly. The *Gata3* threshold is apparently reached at 50% gene dosage in human. Such a threshold, however, is not observed in *Gata3* heterozygous mice [32]. Instead, UGS anomalies in mice are only observed at *Gata3* expression levels below 50% of the wild-type levels [48], complicating the use of *Gata3* mutant mice as disease models. The mosaic inactivation we observe in *Gata3^ND−/−^* embryos may, in fact, better mimic the threshold mechanism of HDR malformations whereby some cells respond normally to *Gata3* while others behave aberrantly. In this respect, it is possible that the urogenital anomalies of HDR patients are caused by premature nephric duct progenitor cell differentiation and involve an aberrant response to GDNF and Fgf signaling. Hence, the *Gata3^ND−/−^* embryos may provide a suitable model to understand the molecular mechanisms and morphogenetic defects underlying HDR syndrome and other CAKUT-related UGS diseases.

## Materials and Methods

### Mice

Conditional and germ-line *Gata3* knockout mice were generated by crossing *Gata3^ex4GFP^* mice [33] with *ACTB-FLPe* [35] and *More-Cre* [36] transgenic mice. The *Gata3^flox^* and *Gata3^Δ^* alleles were genotyped using the primers 5′-TATCAGCGGTTCATCTACAGC and 5′-TGGTAGAGTCCGCAGGCATT. *HoxB7-Cre* and *Rosa26^STOPlacZ^* [38],[54] mice were purchased from The Jackson Laboratory. All mice were kept in a pure C57BL/6 genetic background. Mutant mice for *Ctnnb1* [45] and *Ret* [16] were generated as described. The *HoxB7-Venus* line will be reported elsewhere.

### In Situ Hybridization and Histology

Embryo dissections and processing as well as in situ hybridization on whole mount embryos or tissue sections were performed as described [55],[56]. The *Gata3-exon 4* probe was generated by PCR amplification of the coding sequence of *Gata3* exon 4 and subsequent cloning into the pGEM-T-easy vector. The *Daple* (*Ccdc88c*) in situ probe was transcribed from image clone 6408630 linearized at an internal *Asp718* restriction site. The *Emx2* [57] *Wnt4* [58], *Ret* [59], *Gata3* [60], *Axin2* [61], *Fgf8* [62], *GDNF* [63], *Wnt11* [52], *Slit2* [64], *Spry1* [65] and *Fgf10* [66] in situ probes have been reported previously. Hematoxylin and eosin stainings were performed on 6 µm-thick paraffin sections using standard procedures.

### Immunohistochemistry

Frozen sections have been prepared for immunohistochemistry as described [67]. For immunostainings against β-catenin and Zo1+, an antigen retrieval step has been included [45]. The following antibodies and conjugates were used: rabbit anti-phospho-H3 (1∶200, Upstate Biotechnology), rat anti-E-cadherin (1∶400, Zymed Laboratories), mouse anti-β-catenin (1∶400, Sigma), rabbit anti-phospho-β-catenin (1∶100, Cell signaling), rat anti-Zo1+ (1∶400, Chemicon), rabbit anti-GFP (1∶1000, Abcam) and biotinylated dolichos biflorus agglutinin (DBA) (1∶500, Vector Laboratories). Secondary detection was performed, using Alexa488 or Alexa568 labeled anti-mouse, anti-rabbit or anti-rat antibodies (1∶200, Invitrogen). DBA-lectin staining was visualized with FITC or Cy5 conjugated streptavidin (1∶200, Zymed Laboratories). FgfR2-ligand detection was performed on 12 µm thick cryosections cut from freshly frozen unfixed embryos. After 3 washes in cold PBS, the slides were incubated for 1 hr at 4°C in blocking solution (10% normal goat serum, 2% BSA in PBS supplemented with 0.5 mM MgCl_2_ and 1 mM CaCl_2_). The slides were treated with 2.5 µg/ml mouse FgfR2-Fc (R&D systems) in 0.5× blocking solution for 1 hr at 4°C. Following several washes in PBS, the slides were fixed for 10′ in 4%PFA. Subsequently, the samples were handled as for standard immunohistochemistry. Secondary detection was performed using a successive combination of biotinylated anti-human IgG (1∶500, Vector Laboratories) and streptavidin-FITC conjugate (1∶200, Zymed Laboratories). All slides were counterstained with 50 µg/ml DAPI and mounted with Slow Fade Gold mounting medium (Invitrogen).

### Organ Culture and β-Galactosidase Staining

Mouse urogenital ridges were micro-dissected in cold PBS supplemented with 1% FBS, 1 mM CaCl_2_ and 0.5 mM MgCl_2_. The ridges were collected into 10% FBS/DMEM medium (Wisent) containing Penicillin/Streptomycin (Gibco) and L-Glutamin (Gibco), preincubated at 37°C in 5% CO_2_ and kept under these conditions until all ridges were dissected. Subsequently, the urogenital ridges were cultured in the presence of the appropriate compound on 0.4 µm Transwell filters (Corning) at 37°C in 5% CO_2_ for 42 hrs. The following compounds were used: rat recombinant GDNF (300 ng/ml, R&D systems) anti-GDNF blocking antibody (10 or 20 µg/ml, R&D systems), SU5416 (20 µM, Calbiochem), IgG (20 µg/ml), FgfR2-Fc fusion protein (20 mg/ml, R&D systems) or DMSO (0.01%, Fisher Scientific). β-Galactosidase activity was detected by X-gal staining of dissected or cultured urogenital ridges as described [68].

### Cell Culture

Murine inner medullary collecting duct cells (mIMCD3, kindly provided by Dr Paul Goodyer) were cultured in a 1∶1 mix of DMEM and HAM's F12 media (Wisent) supplemented with 10% fetal bovine serum. To induce the canonical Wnt-pathway, mIMCD3 cells were stimulated with 3 µM BIO ((2′Z,3′E)-6-Bromoindirubin-3′-oxime, EMD) or 0.3% DMSO for 4 hours. RNA was extracted (Rneasy Mini kit, Qiagen) and reverse transcribed (Superscript III, Invitrogen) according to the manufacturer's instructions. Quantitative PCR was performed using iQ Sybr Green Supermix (BioRad) in a RealPlex^2^ cycler (Eppendorf) using the following primers: *Gata3* sense 5′-CTCTGGAGGAGGAACGCTAA-3′ and antisense 5′-TTTGCACTTTTTCGATTTGC-3′, *S16* sense 5′-GTACAAGTTACTGGAGCCTGTTTTG-3′ and antisense 5′-GCCTTTGAGATGGACTGTCGGATGG-3′. For the production of mIMCD3 cells, stably overexpressing Gata3, mouse *Gata3* cDNA was cloned into pMSCV-HA3-IRES-GFP vector (kindly provided by Dr. Jerry Pelletier). The Gata3-pMSCV vector was then co-transfected with pVPack-GP and pVPack-VSV-G vectors (Stratagene) in HEK-293T cells, for virus production. The virus containing supernatant was harvested after 48 hrs by filtration and added to mIMCD3 cells. 48 hours post-infection, the cells were sorted according to GFP expression on an FACSAria-sorter (BD-Bioscience). Gata3 expression levels were assessed by western blotting and the medium level expressing cells were used for the subsequent β-Galactosidase reporter analysis. The conserved *RET* regulatory element was isolated from human RCC cells (ACHN) using published primers [50] and subcloned into the pGEM-T-easy vector (Promega). The point mutation in the Gata3 binding site was introduced via site-specific mutagenesis using the overlap extension method. Subsequently, the wild-type and mutated element were cloned into the β-Galactosidase expression vector pTrap [68] using the unique *SalI*, *SphI* restriction sites. The pGAL4-SV40-Luc vector (kindly provided by Dr. Xiang-Jiao Yang), constitutively expressing firefly luciferase, was used as a transfection control. The Gata3 expressing mIMCD3 were transiently co-transfected with either the wild-type or mutated pTrap vector in combination with pGAL4-SV40-Luc using Lipofectamine 2000 (Invitrogen) according to manufacturer's instructions. The analysis was carried out 24 hours post-transfection with the DualLight Combined Reporter Gene Assay System (Applied Biosystems) according to manufacturer's instructions. All experiments were done in triplicates.

### Bioinformatics

The sequence alignments were performed, using blast2seq (NCBI). The transcription factor binding sites were identified with Mac Vector.

## References

[1] Pohl M, Bhatnagar V, Mendoza SA, Nigam SK (2002). Toward an etiological classification of developmental disorders of the kidney and upper urinary tract.. Kidney Int.

[2] Ichikawa I, Kuwayama F, Pope JCt, Stephens FD, Miyazaki Y (2002). Paradigm shift from classic anatomic theories to contemporary cell biological views of CAKUT.. Kidney Int.

[3] Fedele L, Bianchi S, Agnoli B, Tozzi L, Vignali M (1996). Urinary tract anomalies associated with unicornuate uterus.. J Urol.

[4] Schedl A (2007). Renal abnormalities and their developmental origin.. Nat Rev Genet.

[5] Bouchard M, Souabni A, Mandler M, Neubuser A, Busslinger M (2002). Nephric lineage specification by Pax2 and Pax8.. Genes Dev.

[6] Saxen L, Barlow PW, Green PB, White CC (1987). Organogenesis of the kidney;.

[7] Vize PD, Jones EA, Pfister R (1995). Development of the Xenopus pronephric system.. Developmental Biology.

[8] Dressler GR (2006). The cellular basis of kidney development.. Annu Rev Cell Dev Biol.

[9] Costantini F, Shakya R (2006). GDNF/Ret signaling and the development of the kidney.. Bioessays.

[10] Kuwayama F, Miyazaki Y, Ichikawa I (2002). Embryogenesis of the congenital anomalies of the kidney and the urinary tract.. Nephrol Dial Transplant.

[11] Cacalano G, Farinas I, Wang LC, Hagler K, Forgie A (1998). GFRalpha1 is an essential receptor component for GDNF in the developing nervous system and kidney.. Neuron.

[12] Enomoto H, Araki T, Jackman A, Heuckeroth RO, Snider WD (1998). GFR alpha1-deficient mice have deficits in the enteric nervous system and kidneys.. Neuron.

[13] Moore MW, Klein RD, Farinas I, Sauer H, Armanini M (1996). Renal and neuronal abnormalities in mice lacking GDNF.. Nature.

[14] Pichel JG, Shen L, Sheng HZ, Granholm AC, Drago J (1996). Defects in enteric innervation and kidney development in mice lacking GDNF.. Nature.

[15] Sanchez MP, Silos-Santiago I, Frisen J, He B, Lira SA (1996). Renal agenesis and the absence of enteric neurons in mice lacking GDNF.. Nature.

[16] Schuchardt A, D_Agati V, Larsson_Blomberg L, Costantini F, Pachnis V (1994). Defects in the kidney and enteric nervous system of mice lacking the tyrosine kinase receptor Ret.. Nature.

[17] Grieshammer U, Le M, Plump AS, Wang F, Tessier-Lavigne M (2004). SLIT2-mediated ROBO2 signaling restricts kidney induction to a single site.. Dev Cell.

[18] Kume T, Deng K, Hogan BL (2000). Murine forkhead/winged helix genes Foxc1 (Mf1) and Foxc2 (Mfh1) are required for the early organogenesis of the kidney and urinary tract.. Development.

[19] Miyazaki Y, Oshima K, Fogo A, Hogan BL, Ichikawa I (2000). Bone morphogenetic protein 4 regulates the budding site and elongation of the mouse ureter.. J Clin Invest.

[20] Michos O, Panman L, Vintersten K, Beier K, Zeller R (2004). Gremlin-mediated BMP antagonism induces the epithelial-mesenchymal feedback signaling controlling metanephric kidney and limb organogenesis.. Development.

[21] Basson MA, Akbulut S, Watson-Johnson J, Simon R, Carroll TJ (2005). Sprouty1 is a critical regulator of GDNF/RET-mediated kidney induction.. Dev Cell.

[22] Bertoli-Avella AM, Conte ML, Punzo F, de Graaf BM, Lama G (2008). ROBO2 Gene Variants Are Associated with Familial Vesicoureteral Reflux.. J Am Soc Nephrol.

[23] Lu W, van Eerde AM, Fan X, Quintero-Rivera F, Kulkarni S (2007). Disruption of ROBO2 is associated with urinary tract anomalies and confers risk of vesicoureteral reflux.. Am J Hum Genet.

[24] Skinner MA, Safford SD, Reeves JG, Jackson ME, Freemerman AJ (2008). Renal aplasia in humans is associated with RET mutations.. Am J Hum Genet.

[25] Nakano T, Niimura F, Hohenfellner K, Miyakita E, Ichikawa I (2003). Screening for mutations in BMP4 and FOXC1 genes in congenital anomalies of the kidney and urinary tract in humans.. Tokai J Exp Clin Med.

[26] Weber S, Taylor JC, Winyard P, Baker KF, Sullivan-Brown J (2008). SIX2 and BMP4 mutations associate with anomalous kidney development.. J Am Soc Nephrol.

[27] Patient RK, McGhee JD (2002). The GATA family (vertebrates and invertebrates).. Curr Opin Genet Dev.

[28] Van Esch H, Groenen P, Nesbit MA, Schuffenhauer S, Lichtner P (2000). GATA3 haplo-insufficiency causes human HDR syndrome.. Nature.

[29] Hernandez AM, Villamar M, Rosello L, Moreno-Pelayo MA, Moreno F (2007). Novel mutation in the gene encoding the GATA3 transcription factor in a Spanish familial case of hypoparathyroidism, deafness, and renal dysplasia (HDR) syndrome with female genital tract malformations.. Am J Med Genet A.

[30] Van Esch H, Devriendt K (2001). Transcription factor GATA3 and the human HDR syndrome.. Cell Mol Life Sci.

[31] Lim KC, Lakshmanan G, Crawford SE, Gu Y, Grosveld F (2000). Gata3 loss leads to embryonic lethality due to noradrenaline deficiency of the sympathetic nervous system.. Nat Genet.

[32] Pandolfi PP, Roth ME, Karis A, Leonard MW, Dzierzak E (1995). Targeted disruption of the GATA3 gene causes severe abnormalities in the nervous system and in fetal liver haematopoiesis.. Nat Genet.

[33] Grote D, Souabni A, Busslinger M, Bouchard M (2006). Pax2/8-regulated Gata3 expression is necessary for morphogenesis and guidance of the nephric duct in the developing kidney.. Development.

[34] Khandekar M, Suzuki N, Lewton J, Yamamoto M, Engel JD (2004). Multiple, distant Gata2 enhancers specify temporally and tissue-specific patterning in the developing urogenital system.. Mol Cell Biol.

[35] Rodriguez CI, Buchholz F, Galloway J, Sequerra R, Kasper J (2000). High-efficiency deleter mice show that FLPe is an alternative to Cre- loxP.. Nat Genet.

[36] Tallquist MD, Soriano P (2000). Epiblast-restricted Cre expression in MORE mice: a tool to distinguish embryonic vs. extra-embryonic gene function.. Genesis.

[37] Ting CN, Olson MC, Barton KP, Leiden JM (1996). Transcription factor GATA-3 is required for development of the T-cell lineage.. Nature.

[38] Soriano P (1999). Generalized lacZ expression with the ROSA26 Cre reporter strain.. Nat Genet.

[39] Mologni L, Sala E, Cazzaniga S, Rostagno R, Kuoni T (2006). Inhibition of RET tyrosine kinase by SU5416.. J Mol Endocrinol.

[40] Shakya R, Watanabe T, Costantini F (2005). The role of GDNF/Ret signaling in ureteric bud cell fate and branching morphogenesis.. Dev Cell.

[41] Chi L, Zhang S, Lin Y, Prunskaite-Hyyrylainen R, Vuolteenaho R (2004). Sprouty proteins regulate ureteric branching by coordinating reciprocal epithelial Wnt11, mesenchymal Gdnf and stromal Fgf7 signalling during kidney development.. Development.

[42] Qiao J, Bush KT, Steer DL, Stuart RO, Sakurai H (2001). Multiple fibroblast growth factors support growth of the ureteric bud but have different effects on branching morphogenesis.. Mech Dev.

[43] Steinberg Z, Myers C, Heim VM, Lathrop CA, Rebustini IT (2005). FGFR2b signaling regulates ex vivo submandibular gland epithelial cell proliferation and branching morphogenesis.. Development.

[44] Eswarakumar VP, Lax I, Schlessinger J (2005). Cellular signaling by fibroblast growth factor receptors.. Cytokine Growth Factor Rev.

[45] Marose TD, Merkel CE, McMahon AP, Carroll TJ (2008). Beta-catenin is necessary to keep cells of ureteric bud/Wolffian duct epithelium in a precursor state.. Dev Biol.

[46] Jho EH, Zhang T, Domon C, Joo CK, Freund JN (2002). Wnt/beta-catenin/Tcf signaling induces the transcription of Axin2, a negative regulator of the signaling pathway.. Mol Cell Biol.

[47] Takahashi M, Nakamura Y, Obama K, Furukawa Y (2005). Identification of SP5 as a downstream gene of the beta-catenin/Tcf pathway and its enhanced expression in human colon cancer.. Int J Oncol.

[48] Hasegawa SL, Moriguchi T, Rao A, Kuroha T, Engel JD (2007). Dosage-dependent rescue of definitive nephrogenesis by a distant Gata3 enhancer.. Dev Biol.

[49] Meijer L, Skaltsounis AL, Magiatis P, Polychronopoulos P, Knockaert M (2003). GSK-3-selective inhibitors derived from Tyrian purple indirubins.. Chem Biol.

[50] Fisher S, Grice EA, Vinton RM, Bessling SL, McCallion AS (2006). Conservation of RET regulatory function from human to zebrafish without sequence similarity.. Science.

[51] Michael L, Sweeney DE, Davies JA (2007). The lectin Dolichos biflorus agglutinin is a sensitive indicator of branching morphogenetic activity in the developing mouse metanephric collecting duct system.. J Anat.

[52] Majumdar A, Vainio S, Kispert A, McMahon J, McMahon AP (2003). Wnt11 and Ret/Gdnf pathways cooperate in regulating ureteric branching during metanephric kidney development.. Development.

[53] Ali A, Christie PT, Grigorieva IV, Harding B, Van Esch H (2007). Functional characterization of GATA3 mutations causing the hypoparathyroidism-deafness-renal (HDR) dysplasia syndrome: insight into mechanisms of DNA binding by the GATA3 transcription factor.. Hum Mol Genet.

[54] Yu J, Carroll TJ, McMahon AP (2002). Sonic hedgehog regulates proliferation and differentiation of mesenchymal cells in the mouse metanephric kidney.. Development.

[55] Henrique D, Adam J, Myat A, Chitnis A, Lewis J (1995). Expression of a Delta homologue in prospective neurons in the chick.. Nature.

[56] Pearse RV, Scherz PJ, Campbell JK, Tabin CJ (2007). A cellular lineage analysis of the chick limb bud.. Dev Biol.

[57] Yoshida M, Suda Y, Matsuo I, Miyamoto N, Takeda N (1997). Emx1 and Emx2 functions in development of dorsal telencephalon.. Development.

[58] Gavin BJ, McMahon JA, McMahon AP (1990). Expression of multiple novel Wnt-1/int-1-related genes during fetal and adult mouse development.. Genes Dev.

[59] Pachnis V, Mankoo B, Costantini F (1993). Expression of the c-ret proto-oncogene during mouse embryogenesis.. Development.

[60] George KM, Leonard MW, Roth ME, Lieuw KH, Kioussis D (1994). Embryonic expression and cloning of the murine GATA-3 gene.. Development.

[61] Fujimura N, Vacik T, Machon O, Vlcek C, Scalabrin S (2007). Wnt-mediated down-regulation of Sp1 target genes by a transcriptional repressor Sp5.. J Biol Chem.

[62] Crossley PH, Minowada G, MacArthur CA, Martin GR (1996). Roles for FGF8 in the induction, initiation, and maintenance of chick limb development.. Cell.

[63] Srinivas S, Wu Z, Chen CM, D'Agati V, Costantini F (1999). Dominant effects of RET receptor misexpression and ligand-independent RET signaling on ureteric bud development.. Development.

[64] Holmes GP, Negus K, Burridge L, Raman S, Algar E (1998). Distinct but overlapping expression patterns of two vertebrate slit homologs implies functional roles in CNS development and organogenesis.. Mech Dev.

[65] Minowada G, Jarvis LA, Chi CL, Neubuser A, Sun X (1999). Vertebrate Sprouty genes are induced by FGF signaling and can cause chondrodysplasia when overexpressed.. Development.

[66] Bellusci S, Grindley J, Emoto H, Itoh N, Hogan BL (1997). Fibroblast growth factor 10 (FGF10) and branching morphogenesis in the embryonic mouse lung.. Development.

[67] Bouchard M, Pfeffer P, Busslinger M (2000). Functional equivalence of the transcription factors Pax2 and Pax5 in mouse development.. Development.

[68] Pfeffer PL, Bouchard M, Busslinger M (2000). Pax2 and homeodomain proteins cooperatively regulate a 435 bp enhancer of the mouse Pax5 gene at the midbrain-hindbrain boundary.. Development.

[69] Waterman ML, Fischer WH, Jones KA (1991). A thymus-specific member of the HMG protein family regulates the human T cell receptor C alpha enhancer.. Genes Dev.

[70] Orkin SH (1992). GATA-binding transcription factors in hematopoietic cells.. Blood.

